# Update zur Diagnostik der Drittfenstersyndrome

**DOI:** 10.1007/s00106-024-01466-3

**Published:** 2024-05-02

**Authors:** Julia Dlugaiczyk, Sebastian Rösch, Georgios Mantokoudis

**Affiliations:** 1 Klinik für Ohren‑, Nasen‑, Hals- und Gesichtschirurgie & Interdisziplinäres Zentrum für Schwindel und neurologische Sehstörungen, Universitätsspital Zürich (USZ), Universität Zürich (UZH), Rämistrasse 100, 8091 Zürich, Schweiz; 2 Universitätsklinik für Hals‑, Nasen‑, Ohrenkrankheiten der Paracelsus Medizinischen Privatuniversität Salzburg, Uniklinikum Salzburg, Salzburg, Österreich; 3 Klinik und Poliklinik für Hals-Nasen-Ohrenheilkunde, Universitätsklinikum Regensburg, Regensburg, Deutschland; 4 Universitätsklinik für Hals‑, Nasen- und Ohrenkrankheiten (HNO), Kopf- und Halschirurgie, lnselspital Bern, Universität Bern, Bern, Schweiz

**Keywords:** Audiometrie, Syndrom der oberen Bogengangsdehiszenz, Elektrocochleographie, Erweiterter vestibulärer Aquädukt, Vestibulär evozierte myogene Potenziale, Audiometry, Electrocochleography, Enlarged vestibular aqueduct, Superior canal dehiscence syndrome, Vestibular evoked myogenic potentials

## Abstract

**Hintergrund:**

Die Diagnostik von Drittfenstersyndromen stellt in der klinischen Praxis häufig eine Herausforderung dar.

**Ziel der Arbeit:**

Die vorliegende Arbeit gibt einen aktuellen Überblick über diagnostische Optionen bei diesen Krankheitsbildern, mit besonderem Fokus auf das Syndrom der oberen Bogengangsdehiszenz (SCDS), das Syndrom des erweiterten vestibulären Aquädukts (LVAS) und die X‑chromosomale Malformation der Cochlea.

**Material und Methoden:**

Dazu erfolgte eine Literaturrecherche in der Datenbank PubMed bis Dezember 2023 und die Aufarbeitung eigener Fälle.

**Ergebnisse:**

Audiovestibuläre Testverfahren zur Diagnose eines Drittfenstersyndroms werden in der Literatur am häufigsten im Rahmen des SCDS beschrieben. Für vestibulär evozierte myogene Potenziale wurden hier Grenzwerte mit unterschiedlichen Sensitivitäten/Spezifitäten für verschiedene Messparameter definiert. Neuere Entwicklungen umfassen die Anwendung der Elektrocochleographie, der Breitbandtympanometrie, des Video-Kopfimpulstests und des vibrationsinduzierten Nystagmus. Beim LVAS kommen zunehmend genetische Analysen zum Einsatz.

**Schlussfolgerung:**

Die Diagnose eines Drittfenstersyndroms ergibt sich immer aus der Synthese von Symptomen, klinischen Zeichen, apparativen Untersuchungsbefunden und der Bildgebung.

## Abstract (en)

**Background:**

The diagnosis of third window syndromes often poses a challenge in clinical practice.

**Objective:**

This paper provides an up-to-date overview of diagnostic procedures in third window syndromes, with special emphasis on superior canal dehiscence syndrome (SCDS), large vestibular aqueduct syndrome (LVAS), and X-chromosomal malformation of the cochlea.

**Materials and methods:**

A literature search was performed in PubMed up to December 2023. Furthermore, a selection of the authors’ own cases is presented.

**Results:**

Audiovestibular tests for the diagnosis of third window syndromes are most often reported for patients with SCDS in the literature. In this context, cut-off values with different sensitivities and specificities have been defined for different outcome parameters of vestibular evoked myogenic potentials. Current developments include the application of electrocochleography, broadband tympanometry, video head impulse testing, and vibration-induced nystagmus. Genetic analyses are increasingly applied in LVAS.

**Conclusion:**

The diagnosis of third window syndromes is always based on the synthesis of patients’ symptoms, clinical signs, audiovestibular test results, and imaging.

Bei den Drittfenstersyndromen liegt zusätzlich zum ovalen und runden Fenster noch eine weitere Öffnung des knöchernen Labyrinths vor, welche zu einer veränderten Biomechanik und Fluiddynamik im Innenohr führt. Hieraus resultieren pathognomonische audiovestibuläre Symptome und Untersuchungsbefunde, welche zwischen den einzelnen Krankheitsbildern und selbst bei Patient:innen innerhalb eines Krankheitsbildes stark variieren können.

## Aktuelle diagnostische Verfahren

Der vorliegende Artikel gibt eine aktuelle Übersicht zu den diagnostischen Methoden bei Drittfenstersyndromen unter besonderer Berücksichtigung des Syndroms der oberen Bogengangsdehiszenz („superior canal dehiscence syndrome“, SCDS), des Syndroms des erweiterten vestibulären Aquädukts („large vestibular aqueduct syndrome“, LVAS) und der X‑chromosomalen Malformation der Cochlea. Dabei werden neben den gängigen diagnostischen Verfahren (z. B. Reintonaudiometrie, vestibulär evozierte myogene Potenziale [VEMP], Bildgebung) auch Methoden vorgestellt, welche noch keinen flächendeckenden Einsatz in der klinischen Praxis gefunden haben, z. B. Elektrocochleographie (ECochG), Breitbandtympanometrie, Video-Kopfimpulstest (vHIT), vibrationsinduzierter Nystagmus (VIN) und genetische Tests.

## Überblick über Drittfenstersyndrome

**Abb. 1:**
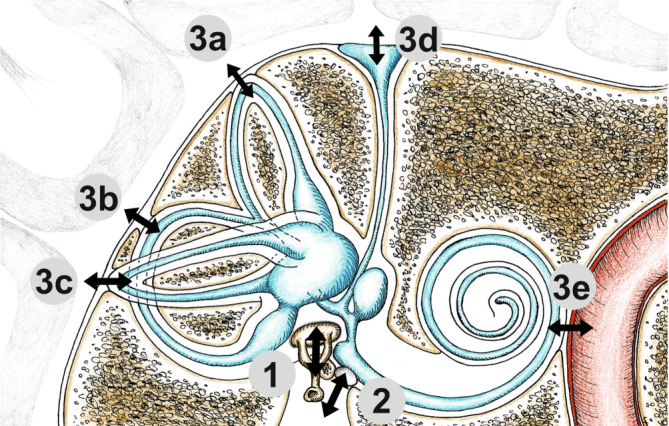
Schematische Darstellung von möglichen Lokalisationen eines dritten Fensters im knöchernen Labyrinth. (Mit freundl. Genehmigung © Dr. med. Hergen Friedrich, alle Rechte vorbehalten.) Neben dem ovalen Fenster (*1*) und dem runden Fenster (*2*) Vorkommen eines zusätzlichen dritten Fensters (*3*) u. a. an folgenden Stellen möglich: superiorer (*3a*), posteriorer (*3b*), lateraler (*3c*) Bogengang, erweiterter vestibulärer Aquädukt (*3d*) oder knöcherne Dehiszenz zwischen Cochlea und Karotiskanal (*3e*)

Eine ausführliche Übersicht hierzu findet sich bei Dlugaiczyk [7]. Zusammengefasst lassen sich Drittfenstersyndrome unterscheiden, welche durch die Erweiterung eines natürlichen neurovaskulären Foramens entstehen (z. B. erweiterter vestibulärer Aquädukt, „enlarged vestibular aqueduct“, EVA; oder „incomplete partition type III“) im Gegensatz zu Syndromen, bei denen zusätzlich eine nicht natürliche Öffnung der otischen Kapsel vorliegt (z. B. Bogengangsdehiszenzen: superior > posterior > lateral in absteigender Häufigkeit; oder knöcherne Dehiszenz zwischen Cochlea und Karotiskanal; Abb. 1).

Es gibt auch diffuse Läsionen, welche zur Schwächung der otischen Kapsel führen können

Während sich der Großteil der Drittfenstersyndrome wie bei den bisher genannten Beispielen auf einen klar lokalisierten Defekt zurückführen lässt, gibt es auch diffuse Läsionen, welche in der Summe zu einer Schwächung der otischen Kapsel im Sinne einer Dehiszenz führen können (z. B. Osteogenesis imperfecta oder fibröse Dysplasie des Schläfenbeins). Außerdem wird zwischen angeborenen und erworbenen Drittfenstersyndromen unterschieden sowie zwischen primären und sekundären. Letztere finden sich insbesondere bei entzündlichen (z. B. Cholesteatom), infektiösen (z. B. Otosyphilis), vaskulären (z. B. Paragangliom) und neoplastischen (z. B. Plasmozytom, Langerhans-Zell-Histiozytose, Tumoren des endolymphatischen Sacks) destruktiven Prozessen des Schläfenbeins.

Durch das zusätzliche „dritte“ Fenster im knöchernen Labyrinth entsteht ein „Locus minoris resistentiae“, welcher in einer komplexen Veränderung der Biomechanik und der Fluiddynamik von Cochlea und Gleichgewichtsorgan resultiert [6, 7, 17]. Die daraus entstehenden pathognomonischen Symptome, klinischen Zeichen und audiovestibulären Untersuchungsbefunde sind in Tab. 1 für das SCDS zusammengefasst, gelten aber auch für andere Drittfenstersyndrome. Besonders ist hier die innenohrbedingte Schallleitungsschwerhörigkeit im Reintonaudiogramm (RTA) mit teilweise negativen Knochenleitungsschwellen und einem „air-bone gap“ (ABG) mit maximaler Ausprägung zwischen 250 und 1000 Hz zu erwähnen [24].

**Tab. 1:** Kriterien gemäß International Classification of Vestibular Disorders (ICVD) für das Syndrom der oberen Bogengangsdehiszenz (SCDS) [40]

**Abb. 2:**
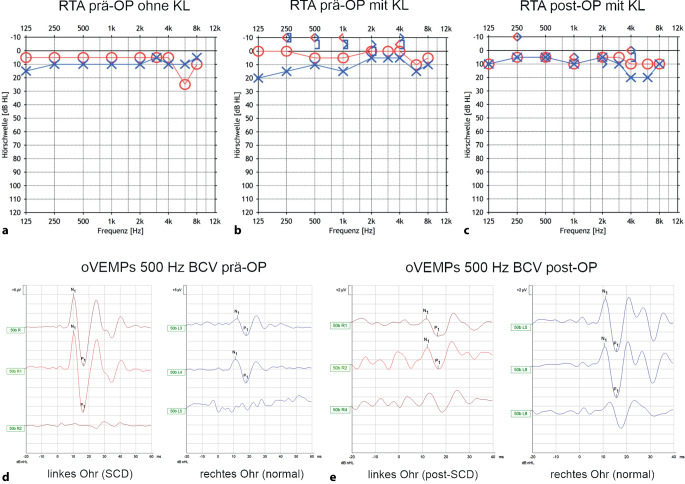
Präoperative (**a**, **b**, **d**) und postoperative (**c**, **e**) audiovestibuläre Befunde einer Patientin mit einem Syndrom der oberen Bogengangsdehiszenz (SCDS) links. Verschluss der Dehiszenz durch transmastoidales Plugging. **a** Präoperatives Reintonaudiogramm (RTA) ohne Knochenleitungsschwellen: normale Luftleitungsschwelle bds. **b** Präoperatives RTA mit Luft- und Knochenleitungsschwellen: negative Knochenleitungsschwellen bds. (Erläuterung s. Text). **c** Postoperatives RTA mit Normalisierung der Knochenleitungsschwellen. **d** Präoperative okuläre vestibulär evozierte myogene Potenziale (oVEMP) bei 500 Hz Knochenleitungsstimulus (BCV) an Fz (Mitte der Stirn am Haaransatz). oVEMP-n10p15-Amplitude (N1P1) von durchschnittlich 40 µV für das linke Ohr (erhöht), und 10 µV für das rechte Ohr (normal). Maßstab *y‑Achse* 5 μV. **e** Normalisierung der oVEMP-n10p15-Amplitude (N1P1) links postoperativ (3 μV) bei gleichbleibender Amplitude rechts (10 μV). Maßstab *y‑Achse* 2 μV. *SCD* obere Bogengangsdehiszenz

**1) ≥** **1 Symptom, welches mit einem „dritten Fenster“ des Innenohrs vereinbar ist**
Hyperakusis für Knochenleitungsreize (*Autophonie, Hören von körpereigenen Geräuschen im betroffenen Ohr, z.* *B. Augenbewegungen, Darmgeräusche, Schritte*)
Schallinduzierter Schwindel bzw. Oszillopsien mit zeitlicher Koppelung an den Stimulus (*Tullio-Phänomen)*
Druckinduzierter Schwindel bzw. Oszillopsien mit zeitlicher Koppelung an den Stimulus
Pulsatiler Tinnitus
**2) ≥** **1 der folgenden klinischen Zeichen oder Untersuchungsbefunde, welche für ein „drittes Fenster“ im Innenohr sprechen**
Exzitatorischer oder inhibitorischer Nystagmus in der Ebene des betroffenen oberen Bogengangs, ausgelöst durch Schall *(a. e. ≥* *100* *dB nHL) *oder Änderungen des Mittelohr- bzw. intrakraniellen Drucks *(z.* *B. Valsalva-Versuch, Hennebert-Zeichen, Politzer-Test)*
Negative Knochenleitungsschwellen in der Reintonaudiometrie (*typisch für Frequenzen* *≤* *1000* *Hz, häufig auch erhöhte Luftleitungsschwellen in diesem Frequenzbereich →* *innenohrbedingte Schallleitungsschwerhörigkeit; *Abb. 2b)
Gesteigerte VEMP-Antworten (erniedrigte cVEMP-Schwellen und/oder erhöhte oVEMP-Amplituden; Abb. 2d)
**3) Nachweis der Dehiszenz des oberen Bogengangs im hochauflösenden Schläfenbein-CT mit multiplanarer Rekonstruktion** (*empfohlene Schichtdicke ≤* *0,2* *mm; auch DVT möglich; Rekonstruktion in Pöschl-Ebene (parallel zum oberen Bogengang) und Stenvers-Ebene (senkrecht dazu)*)
**4) Nicht besser durch eine andere vestibuläre Erkrankung zu erklären**

*c* zervikal, *CT* Computertomogramm, *DVT* digitale Volumentomographie, *nHL* „normal hearing level“, *o* okulär, *VEMP* „vestibular evoked myogenic potential“

Ergänzungen der Autoren zum Originaltext der Klassifikation sind in kursiver Schrift angegeben

Bestimmen Sie im RTA die Knochenleitungsschwellen bis hin zu negativen Werten.

Unterbleibt diese Messung bei V. a. ein Drittfenstersyndrom, resultiert ein vermeintlich „normales“ Tonaudiogramm (Abb. 2a), und der differenzialdiagnostisch wichtige Hinweis der negativen Knochenleitungsschwellen (Abb. 2b) geht verloren. Dabei ist zu beachten, dass auch bei einem einseitigen SCDS die Knochenleitungsschwellen der kontralateralen (gesunden) Seite negativ sein können aufgrund der Schwierigkeiten der Vertäubung in dieser Situation [40].

Nach dem chirurgischen Verschluss des dritten Fensters sind die normale Biomechanik und Fluiddynamik des Innenohrs wiederhergestellt, was zu einer Rückbildung der klinischen Symptome/Zeichen und einer Normalisierung der pathognomonischen Untersuchungsbefunde führt (Abb. 2c, e; [7, 9, 17]).

## Syndrom der oberen Bogengangsdehiszenz

Die Diagnose eines SCDS erfolgt nach den Kriterien der ICVD (International Classification of Vestibular Disorders) aus der Synthese von charakteristischen klinischen Symptomen, pathognomonischen elektrophysiologischen Befunden und dem radiologischen Nachweis einer knöchernen Dehiszenz des oberen Bogengangs („superior canal dehiscence“, SCD) in der hochauflösenden Computertomographie (CT) bzw. in der digitalen Volumentomographie (DVT) des Schläfenbeins (Tab. 1; [40]). Da sowohl das Vorhandensein als auch die Größe einer SCD in der Bildgebung häufig überschätzt werden, sind weitere diagnostische Tests mit einer hohen Spezifität notwendig, um diejenigen Patient:innen zu identifizieren, welche tatsächlich unter einem klinisch relevanten *Syndrom* der oberen Bogengangsdehiszenz (SCDS) leiden und von einer chirurgischen Therapie profitieren können [26].

### Vestibulär evozierte myogene Potenziale

Die Untersuchungsmethode der vestibulär evozierten myogenen Potenziale (VEMP) wird seit gut 20 Jahren in der Diagnostik von SCDS und anderen Drittfenstersyndromen verwendet. Entsprechend zahlreich sind Studien mit verschiedenen Elektrodenlokalisationen (zervikale [c-] und okuläre [o-]VEMP), Stimulusqualitäten (Luft- und Knochenleitungsreize), Stimulusparametern (Stimulusfrequenz, Morphologie, Repetitionsrate), Messparametern (VEMP-Amplituden, -Latenzen, -Schwellen) sowie Aussagen zur diagnostischen Genauigkeit verschiedener Cut-off-Werte. Eine Zusammenstellung und Diskussion dieser Ergebnisse findet sich u. a. bei Noij und Rauch [26].

Generell liegen bei SCDS gesteigerte VEMP-Antworten vor (erhöhte Amplituden, erniedrigte Schwellen)

Generell liegen bei SCDS gesteigerte VEMP-Antworten vor (erhöhte Amplituden, erniedrigte Schwellen). Ein Beispiel findet sich in Abb. 2d, die pathophysiologischen Grundlagen werden bei [6] und [17] beschrieben. Zusammenfassend lassen sich folgende Erkenntnisse zur VEMP-Diagnostik bei SCDS festhalten, weitere Details finden sich in Tab. 2:

1. oVEMP-Amplituden bei 500 Hz Luftleitungsstimulus („air-conducted sound“, ACS) besitzen eine bessere diagnostische Trennschärfe für das Vorliegen eines SCDS als cVEMP-Schwellen für Click-Stimuli – bei kürzerer Messdauer und geringerer Lärmbelastung [18, 40, 45].

2. Das Vorhandensein von VEMP-Antworten bei hochfrequenten Stimuli (z. B. cVEMP bei 2 kHz, oVEMP bei 4 kHz) ist ein starker Hinweis auf das Vorliegen eines SCDS. Insbesondere die Bestimmung der oVEMP-n10-Amplitude bei 4 kHz gilt als schneller und zuverlässiger „single-step test“. Bei diesen hohen Frequenzen werden im intakten knöchernen Labyrinth i. d. R. keine VEMP-Antworten ausgelöst [5, 21, 25, 35].

Die Bestimmung der oVEMP-Amplitude bei 4 kHz ist ein schneller und zuverlässiger „single-step test“

3. Ist eine hochfrequente VEMP-Messung nicht möglich, so wird zur Verbesserung der diagnostischen Genauigkeit empfohlen, mehrere Messparameter bei niedrigeren Frequenzen zu kombinieren, z. B. in Form des „third window indicator“ (TWI = cVEMP-Schwelle bei 500 Hz ACS – ABG bei 250 Hz). Bei 2 kHz Stimulusfrequenz bringt der TWI keinen zusätzlichen Gewinn mehr gegenüber der Schwellenbestimmung allein ([25]; Tab. 2).

4. Es wurden Cut-off-Werte für verschiedene VEMP-Parameter vorgeschlagen (Tab. 2). Diese hängen von mehreren Faktoren ab (Stimulusparameter, diagnostische Kriterien für SCDS, Kontrollpopulation) und sollten für jedes Vestibularislabor alterskorrigiert bestimmt werden [18, 45]. Generell wird empfohlen, Stimuli mit kurzer Anstiegsflanke („rise time“) zu verwenden, um möglichst hohe VEMP-Amplituden zu erzielen [5].

5. Trotz hoher Sensitivität und Spezifität bieten erhöhte VEMP-Antworten allein meist keine 100%ige diagnostische Sicherheit für das Vorliegen eines SCDS – gerade bei der Unterscheidung zwischen SCDS und anderen Schwindelsyndromen (Tab. 2). Mögliche Differenzialdiagnosen für erhöhte VEMP-Antworten umfassen z. B. andere Drittfenstersyndrome, intravestibuläre Schwannome oder M. Menière im Reizstadium [7].

**Tab. 2:** Literaturübersicht zu Grenzwerten verschiedener o‑ und cVEMP-Parameter für die Diagnose des Syndroms der oberen Bogengangsdehiszenz (SCDS)

Studie	SCDS-Definition	Kontrollgruppe	Mess- und Stimulus-Parameter	Cut-off-Wert	Sensitivität (%)	Spezifität (%)
*oVEMP-Amplitude (500* *Hz ACS)*						
Janky et al. [18]	Intraoperative Bestätigung	Neurootologisch Gesunde (alterskorrigiert)	oVEMP-n10-Ampl. 4 ms Stimulus (1/2/1)^a^ 125 dB SPL	> 8,25 µV	100	100
Zuniga et al. [45]	Intraoperative Bestätigung	Neurootologisch Gesunde (alterskorrigiert)	oVEMP-n10p15-Ampl. 4 ms Stimulus (1/2/1) 125 dB SPL	> 17,1 µV	100	98
Verrecchia et al. [37]	Klinisch + elektrophysiologisch + CT	Patient:innen mit anderen vestibulären Syndromen	oVEMP-n10p15-Ampl. 4 ms Stimulus (1/2/1) 125 dB nHL	>16,7 μV	100	89
*oVEMP-Amplitude (500* *Hz BCV)*						
Manzari et al. [22]	Klinisch + CT	Neurootologisch Gesunde	oVEMP-n10-Ampl. 7 ms Stimulus (1/5/1) 130 dB FL an Fz^b^	> 10 μV	100	97
*oVEMP-Amplitude (4* *kHz ACS)*						
Manzari et al. [21]	Klinisch + CT	Neurootologisch Gesunde	oVEMP-n10-Ampl. 7 ms Stimulus (1/5/1) 120 dB SPL	> 0 μV	100	100
Tran et al. [35]	Ward-Kriterien (2017) [39]	Patient:innen eines Schwindelzentrums	oVEMP-n10p15-Ampl. 5 ms Stimulus (2/1/2) 95 dB nHL	> 0 μV	86,5	87,7
Tran et al. [35]	Ward-Kriterien (2017) [39]	Patient:innen eines Schwindelzentrums	oVEMP-n10p15-Ampl. 5 ms Stimulus (2/1/2) 95 dB nHL	> 15 μV	83,8	96,8
*oVEMP-Amplitude (4* *kHz BCV)*						
Manzari et al. [21]	Klinisch + CT	Gesunde	oVEMP-n10-Ampl. 7 ms Stimulus (1/5/1) 130 dB FL an Fz^b^	> 0 μV	100	100
*cVEMP-Schwellen (500* *Hz ACS)*						
Noij et al. [25]	Klinisch + CT	Neurootologisch Gesunde	cVEMP-Schwelle „2 cycle rise and fall times“ (4/0/4)	< 98 dB peSPL	52	100
Noij et al. [25]	Klinisch + CT	Neurootologisch Gesunde	500 Hz TWI (= cVEMP-Schwelle bei 500 Hz ACS–ABG bei 250 Hz)	< 103 dB	88	100
Tran et al. [35]	Ward-Kriterien (2017)	Patient:innen eines Schwindelzentrums	cVEMP-Schwelle 5 ms Stimulus (2/1/2)	< 75 dB nHL	55,6	96
*cVEMP-Schwelle (2* *kHz ACS)*						
Noij et al. [25]	Klinisch + CT	Neurootologisch Gesunde	cVEMP-Schwelle „2 cycle rise and fall times“ (1/0/1)	< 118 dB peSPL	92	100
Noij et al. [25]	Klinisch + CT	Neurootologisch Gesunde	2 kHz TWI (= cVEMP-Schwelle bei 2 kHz ACS–ABG bei 250 Hz)	<92 dB	92	100

^a^ („rise time/plateau/fall time“) des Stimulus, Dauer jeweils in ms

^b^ Fz: Mitte der Stirn am Haaransatz

*ABG* „air-bone gap“, *ACS* „air-conducted sound“, *Ampl.* Amplitude, *BCV* „bone-conducted vibration“, *CT* Computertomographie, *Fz* Mitte der Stirn am Haaransatz, *FL* „force level“, *nHL* „normal hearing level“, *o-*/*cVEMPs* okuläre/zervikale vestibulär evozierte myogene Potenziale, *(pe)SPL* (Spitzen‑)Schalldruckpegel, *TWI* „third window indicator“

Viele der hier beschriebenen Befundkonstellationen lassen sich auch bei anderen Drittfenstersyndromen beobachten, im Vergleich zum SCDS liegen hier aber viel weniger Informationen aus der Literatur vor.

### Elektrocochleographie

Die Elektrocochleographie (ECochG) misst die elektrischen Potenziale, welche durch akustische Stimulation in der Cochlea generiert werden. Dabei wird das Ohr akustisch über einen Einsteckhörer stimuliert (Clicks 100 μs Dauer, 85 dB *nHL,* „normal hearing level“, oder lange „tone bursts“, 8 ms Dauer) und die elektrische Aktivität der Cochlea über eine Promontorialelektrode (Nadelelektrode) oder über eine Oberflächenelektrode am Trommelfell aufgezeichnet. Die Signale werden 1000- bis 1500-mal gemittelt analog zur Hirnstammaudiometrie. Dieser Test kann entweder in Lokalanästhesie oder in Vollnarkose durchgeführt werden. Die ECochG dient als Überbegriff für das Aufzeichnen verschiedener Signalkomponenten wie Hörnervenneurophone („auditory nerve neurophonics“), cochleäre Mikrophonpotenziale („cochlear microphonics“), Summationspotenziale (SP) und Aktionspotenziale (AP). Die ECochG wurde zur Diagnose des M. Menière schon in den 1980er-Jahren durch William Gibson propagiert [13] und wird seither verwendet [11, 16, 23, 34].

Für die Diagnostik des SCDS werden wie beim M. Menière das SP-Signal (proportional zur Auslenkung der Basilarmembran und Reizung der Nervenfasern [43]) und das AP-Signal (Potenzial des N. cochlearis) verwendet. Die Latenzen und Amplituden variieren mit der Reizstärke [10]: Die Amplituden nehmen mit zunehmender akustischer Reizung proportional zu, während die Latenzen kürzer werden. ECochG-Signale variieren ebenfalls mit dem intracochleären Druck, was auch intraoperativ während eines Valsalva-Manövers gezeigt werden konnte [12]. Veränderungen des Verhältnisses zwischen der SP- und der AP-Amplitude geben Hinweise für eine Fistel respektive für ein drittes Fenster: Ein Missverhältnis ist in 76,5 % [41] bis 89 % [1] der SCDS-Fälle zu erwarten. Die Patient:innen zeigten im Mittel ein SP/AP-Verhältnis von 0,62 bei einem Grenzwert von > 0,4. Die Spezifität liegt bei 70 % mit Feinschicht-CT als Goldstandard ohne Berücksichtigung von Beinahe-Dehiszenzen („near-dehiscence syndrome“; [3]) und Klinik eines Drittfenstersyndroms. Die angegebenen Grenzwerte sind je nach Messaufbau unterschiedlich und müssen vom jeweiligen Anwender an gesunden Probanden ermittelt werden. Eine mögliche Erklärung für die Erhöhung der SP/AP-Ratio beim SCDS – analog zum Endolymphhydrops bei M. Menière – ist ein reduzierter Perilymphdruck aufgrund der knöchernen Dehiszenz, was zu einem relativen Überdruck der Endolymphe führen kann („hydrops e vacuo“) [41].

**Abb. 3:**
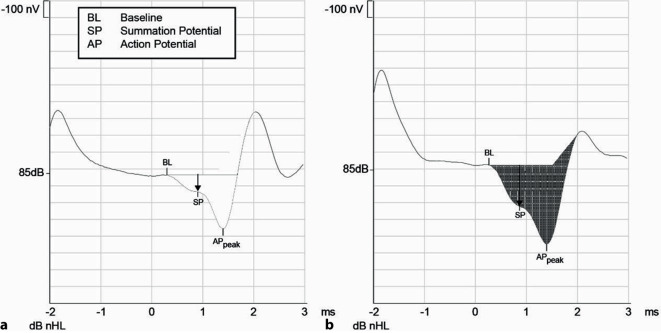
Elektrocochleographische Bestimmung der Amplituden für Summations- (*SP*) und Aktionspotenziale (*AP*) bei einem Luftleitungsstimulus von 85 dB nHL („normal hearing level“). **a** Normalbefund: SP/AP = 0,32. **b** Ohr mit Syndrom der oberen Bogengangsdehiszenz (SCDS): SP/AP = 0,52; *AP peak*: Gipfel des Aktionspotentials

Die Abb. 3a zeigt eine ECochG-Aufzeichnung eines gesunden Probanden mit normalen Amplitudenverhältnissen (SP/AP). Das Verhältnis zwischen dem Summations- und dem Aktionspotenzial wird errechnet (0,32). Dabei werden als Werte die Amplituden oder die Flächen unter der Kurve verwendet. Bei einem Patienten mit SCDS (Abb. 3b) ist ein pathologisches SP/AP-Verhältnis > 0,4 (0,52) zu sehen. Ein SP/AP-Verhältnis > 0,4 in der ECochG unterstützt also die Diagnose eines SCDS.

Ein SP/AP-Verhältnis > 0,4 in der ECochG unterstützt die Diagnose eines SCDS

Die ECochG kann nebst der Diagnostik auch als intraoperatives Monitoring dienen [1, 41], da sich das Signal nach Verschluss der Dehiszenz (Deckung oder Plugging) verändert respektive sich normalisiert [2].

Allerdings besteht noch eine fehlende Standardisierung in der Anwendung des Tests. In über 20 % der Fälle kann kein intraoperatives ECochG-Signal gemessen werden [29]. Dies kann an verschiedenen Faktoren liegen, wie z. B. elektromagnetischen Störungen, Flüssigkeit im Gehörgang, abgekipptem Einsteckkopfhörer, Mittelohrflüssigkeit und Elektrodenplatzierung [29, 30]. Auch besteht eine große Varianz der Normwerte, weshalb sich dieser Test noch nicht im klinischen Alltag durchgesetzt hat. Diese Varianz ist nicht nur abhängig von Platzierung und Art der Elektrode, sondern auch vom gewählten Stimulus, vom Bandfilter (beispielsweise 3,2 Hz zu 3 kHz) und von der vorhandenen Innenohrfunktion [11]. Da die ECochG je nach verwendeter Elektrode semi-invasiv ist, sind viele Fachärzt:innen mit der Anwendung zurückhaltend, weshalb sie in den jetzigen diagnostischen Kriterien der Bárány-Gesellschaft [40] noch nicht abgebildet ist. Nicht chirurgisch tätige HNO-Ärzt:innen sowie Neurolog:innen, welche sich mit Schwindelpatient:innen beschäftigen, bevorzugen häufig nichtinvasive Methoden wie c/oVEMP für die weiterführende elektrophysiologische Diagnostik zur Objektivierung einer Fistel oder eines Drittfenstersyndroms. Nichtsdestotrotz ist eine ECochG-Untersuchung eine wichtige Zusatzuntersuchung, um ein Drittfenstersyndrom zu bestätigen, in Anbetracht der vielen möglichen Differenzialdiagnosen.

### Breitbandtympanometrie

Bei der Breitbandtympanometrie („wideband tympanometry“) wird ein Luftleitungsreiz mit einem Frequenzspektrum von 226–8000 Hz auf das Trommelfell appliziert und die Schallabsorption durch das Mittel‑/Innenohr für die einzelnen Frequenzen bestimmt. Entsprechend der zugrunde liegenden Pathophysiologie beim SCDS mit einer erniedrigten Impedanz des Innenohrs für Frequenzen < 1000 Hz wurde bei SCDS-Patient:innen eine vermehrte Schallabsorption in diesem Bereich gemessen. Zusätzlich ist die Resonanzfrequenz zu niedrigeren Frequenzen verschoben. Nach erfolgreichem operativem Verschluss der Dehiszenz normalisieren sich diese Befunde [36]. Da bisher nur geringe Patientenzahlen publiziert wurden, lässt sich die Sensitivität und Spezifität dieser Methode für die Diagnose eines SCDS bzw. eines Drittfenstersyndroms bisher nicht beurteilen. Weitere Studien sind notwendig, um den zusätzlichen Nutzen im klinischen Alltag zu beurteilen [9].

### Video-Kopfimpulstest

In den letzten Jahren wurde bei Patient:innen mit SCDS über eine isolierte Unterfunktion (d. h. reduzierter Gain + Korrektursakkaden) des betroffenen superioren Bogengangs im Video-Kopfimpulstest (vHIT) berichtet. Dieser Befund ist sicher nützlich bei der Abgrenzung des SCDS von anderen Differenzialdiagnosen. Als mögliche zugrunde liegende Pathomechanismen für die Gain-Reduktion werden ein (inkomplettes) Auto-Plugging des betroffenen Bogengangs durch die darüberliegende Dura oder ein Verlust von Strömungsenergie der Endolymphe über die Dehiszenz („endolymphatic flow dissipation“) diskutiert [4].

### Vibrationsinduzierter Nystagmus

Der sehr einfach und schnell durchzuführende Test des vibrationsinduzierten Nystagmus (VIN) ist sehr sensitiv für die Detektion eines SCDS, wird aber in der klinischen Praxis noch eher selten angewandt. Zusammengefasst nutzt dieser „vestibuläre Weber-Test“ die erhöhte Sensitivität des Innenohrs für Knochenleitungsreize bei SCDS. Appliziert man einen Vibrationsreiz mit einer Stimulusfrequenz zwischen 60 und 800 Hz auf dem Vertex, so wird bei 88 % der Patient:innen mit SCDS ein stimulusgekoppelter Nystagmus ausgelöst, welcher in 95 % der Fälle in Richtung des betroffenen Ohrs schlägt, das Maximum der Antwort liegt bei etwa 400 Hz. Entsprechend dem ersten Ewald-Gesetz zeigt sich durch die Stimulation des superioren Bogengangs ein überwiegend vertikal-torsioneller Nystagmus. Ein Überblick über die Methodik und die Anwendung des VIN bei SCDS findet sich bei Dumas et al. [8], die neurophysiologischen Grundlagen werden bei Curthoys et al. [6] erläutert.

## Syndrom des erweiterten vestibulären Aquädukts

Ein erweiterter vestibulärer Aquädukt (EVA) stellt eine anatomische Variante im Bereich des dorsalen Felsenbeins dar, welche aufgrund ihrer irregulären Vergrößerung in unterschiedlichen bildgebenden Verfahren erkennbar wird ([32]; Abb. 1 und 4a). Nach den Cincinnati-Kriterien wird ein EVA in der axialen CT des Schläfenbeins folgendermaßen definiert: Durchmesser knöcherner Aquaeductus vestibuli > 1,9 mm (Operculum) bzw. > 0,9 mm (Mitte zwischen Vestibulum und Operculum) [38].

Bei Vorliegen eines EVA mit klinischen Symptomen wird auch von einem Syndrom des erweiterten vestibulären Aquädukts (LVAS) gesprochen. Als Ursache für die atypische Morphologie des knöchernen Kanals (Aquädukt) und des darin befindlichen Ductus endolymphaticus werden Störungen der pH-Wert- und Volumenregulation während der Embryogenese angesehen [14], welche sekundär zu Veränderungen der umgebenden Strukturen führen. Ein EVA wird als eine der häufigsten Malformationen des Felsenbeins beschrieben [42], ist aber keinem spezifischen Krankheitsbild zuzuordnen. Vielmehr stellt er einen klinischen Hinweis für eine mögliche genetische Assoziation eines vorliegenden Krankheitsbildes dar [27].

**Abb. 4:**
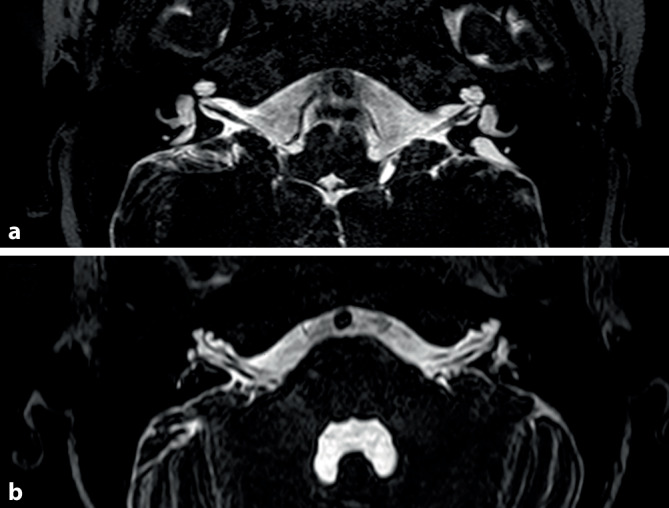
T2-gewichtete Magnetresonanztomographie der Kleinhirnbrückenregion zweier unterschiedlicher Patienten (**a** und **b**). Axiale Schichtung. (Aus [27] © S. Rösch et al., CC BY 4.0, https://creativecommons.org/licenses/by/4.0/.) **a** Patient mit beidseits deutlich erweitertem vestibulärem Aquädukt, dorsal des angeschnittenen horizontalen Bogengangs. **b** Patient mit beidseitiger X‑chromosomaler Malformation der Cochlea mit fehlendem Modiolus („incomplete partition type III“)

Der EVA ist eine der häufigsten Malformationen des Felsenbeins

Varianten im *SLC26A4-*Gen stellen dabei die häufigste genetische Ursache dar, sind aber bei EVA-Patient:innen asiatischen Ursprungs deutlich häufiger nachweisbar als bei europäischen (kaukasischen) Patient:innen [27]. Weitere Gene, deren Varianten in Zusammenhang mit einem EVA in kaukasischen Patientenkohorten wiederholt beschrieben wurden, sind *FOXI1, KCNJ10, POU3F4, GJB2, TMC1 *[27]*, CHD7* [28] sowie ein haploider Genotyp (Haplotyp) stromaufwärts des *SLC26A4*-Gens [15]. Die Vielzahl möglicher ursächlicher Gene führt zu entsprechend unterschiedlichen klinischen Ausprägungen bzw. Phänotypen.

Bei Vorliegen eines EVA immer an Schilddrüsenabklärung und genetische Diagnostik denken

Ein EVA wird sowohl bei nichtsyndromaler Schwerhörigkeit mit EVA (DFNB4) als auch bei syndromalen Formen wie dem Pendred-Syndrom, der distalen renal-tubulären Azidose mit Schwerhörigkeit, dem CHARGE-Syndrom (Kolobom, Herzfehler, Atresia choanae, Retardierung von Wachstum und Entwicklung, genitale Anomalien, Ohrfehlbildungen), dem Waardenburg-Syndrom, dem branchiootorenalen Syndrom und dem branchiookulofazialen Syndrom beschrieben. Eine genetische Abklärung zur möglichen Ursachenklärung, eine gezielte Beratung sowie eine frühzeitige Erkennung möglicher weiterer Befunde [19] sind daher bei Vorliegen eines EVA in Betracht zu ziehen. Zusätzlich sollte eine Schilddrüsenabklärung erfolgen (Pendred-Syndrom). 

Das klinische Bild bei EVA wird in Analogie zu den Ursachen sehr divers beschrieben, sowohl hinsichtlich audiologischer als auch vestibulärer Befunde [15, 20, 44]. Hörstörungen treten häufig prä- oder perilingual auf und sind i. d. R. progredient. Bezüglich Form der Schwerhörigkeiten wird von Schallleitungs‑, Schallempfindungs- oder kombinierten Schwerhörigkeiten berichtet. Hierzu tragen möglicherweise mehrere Aspekte bei. Neben dem Zeitpunkt einer Untersuchung – frühe Form einer Hörstörung mit nur geringem Ausmaß und möglichweise noch sichtbarer Schallleitungskomponente – sind auch die angewandten audiometrischen Verfahren entscheidend für die Kategorisierung einer Hörstörung, gerade bei Kleinkindern. Die fachgerechte Messung der Knochenleitung ist maßgeblich für die Erkennung und Bewertung eines möglichen ABG als Teil eines Drittfensterphänomens. Schließlich wird ein EVA unter Umständen erst bei ausgeprägter Innenohrschwerhörigkeit im Rahmen einer präoperativen Bildgebung vor Cochleaimplantation erkannt und erlaubt erst zeitlich verzögert die Zuordnung präexistenter Symptome zu einem Drittfenstersyndrom.

Vestibuläre Störungen werden seltener bei EVA-Patient:innen beschrieben. Auch diesbezüglich sind klinische Berichte sehr unterschiedlich. Sowohl die VEMP-Messungen [44] als auch vHIT-Untersuchungen [20] zeigen sich im Fall von EVA-Patient:innen häufig auffällig. Die inkongruenten Ergebnisse zwischen Kalorik und vHIT gleichen dabei denen von Patient:innen mit endolymphatischem Hydrops (M. Menière) [33].

## X-Chromosom-assoziierte Malformation der Cochlea

Die durch das Fehlen des Modiolus radiologisch auffällige Malformation (Abb. 4b), auch „incomplete partition type III“ genannt [31], stellt eine weitere anatomische Variante dar, welche durch die abnorme Erweiterung des inneren Gehörgangs und die inkomplette knöcherne Trennung zwischen Cochlea und innerem Gehörgang ursächlich für ein Drittfensterphänomen sein kann. Die X‑chromosomal bedingte Malformation ist häufig mit Mutationen des *POUF3F4*-Gens assoziiert, betrifft überwiegend männliche Patienten und geht in etwa 50 % der Fälle mit einem EVA auf der gleichen Seite einher [27]. Aufgrund der Assoziation mit einem Perilymph-Gusher bei Eröffnung der otischen Kapsel wird das Syndrom auch X‑chromosomale Schwerhörigkeit mit Stapes-Gusher (*DFNX2*) genannt [7].

Die klinische Symptomatik bzgl. audiologischer und vestibulärer Auffälligkeiten ist ähnlich der eines EVA. Erschwerend für die Interpretation audiologischer Befunde und die darauf basierende Therapieentscheidung ist eine häufig begleitende Fixierung der Steigbügelfußplatte, welche ebenfalls zu einer Schallleitungskomponente beitragen kann.

## Fazit für die Praxis

- Die Diagnose eines Drittfenstersyndroms ergibt sich immer aus der Synthese von Symptomen, klinischen Zeichen, apparativen Untersuchungsbefunden und der Bildgebung.
- Achten Sie bei der Computertomographie (CT)/digitalen Volumentomographie (DVT) des Schläfenbeins auf eine ausreichend dünne Schichtdicke (ideal ≤ 0,2 mm) und auf eine multiplanare Rekonstruktion (bei V. a. Syndrom der oberen Bogengangsdehiszenz, SCDS, in der Pöschl- und Stenvers-Ebene).
- Schauen Sie die Bildgebung immer selbst an – auch über den superioren Bogengang hinaus!
- Denken Sie beim Vorliegen eines erweiterten vestibulären Aquädukts (EVA) an weitere Innenohrfehlbildungen und eine mögliche Beteiligung anderer Organe (Pendred-Syndrom!).

## Abkürzungen

ABG: „Air-bone gap“
ACS: Luftleitungsschall („air-conducted sound“)
AP: Aktionspotenzial
BCV: Knochenleitungsvibration („bone-conducted vibration“)
c: Zervikal
CT: Computertomographie
DVT: Digitale Volumentomographie
ECochG: Elektrocochleographie
EVA: Erweiterter vestibulärer Aquädukt („enlarged vestibular aqueduct“)
FL: „Force level“
ICVD: International Classification of Vestibular Disorders
LVAS: Syndrom des erweiterten vestibulären Aquädukts („large vestibular aqueduct syndrome“)
nHL: „Normal hearing level“
o: Okulär
pe: Spitzen- („peak“)
RTA: Reintonaudiogramm
SCD: Obere Bogengangsdehiszenz („superior canal dehiscence“)
SCDS: Syndrom der oberen Bogengangsdehiszenz („superior canal dehiscence syndrome“)
SP: Summationspotenzial
SPL: Schalldruckpegel („sound pressure level“)
TWI: „Third window indicator“
VEMP: Vestibulär evozierte myogene Potenziale
vHIT: Video-Kopfimpulstest („video head impulse test“)
VIN: Vibrationsinduzierter Nystagmus
